# A novel cuproptosis-related gene signature for overall survival prediction in patients with hepatocellular carcinoma

**DOI:** 10.1016/j.heliyon.2022.e11768

**Published:** 2022-11-21

**Authors:** Fan Yang, Shitao Jiang, Yaoge Liu, Ting Zhang, Chengpei Zhu, Lei Zhang, Xinting Sang, Xin Lu, Jiaxin Wei, Kaige Deng, Yongchang Zheng, Yiyao Xu

**Affiliations:** a Department of Liver Surgery, State Key Laboratory of Complex Severe and Rare Diseases, Peking Union Medical College Hospital, Chinese Academy of Medical Sciences and Peking Union Medical College, Beijing, China; b Department of Emergency, Peking Union Medical College Hospital, Chinese Academy of Medical Sciences and Peking Union Medical College, Beijing, China

**Keywords:** Cuproptosis, Hepatocellular carcinoma, Immune, Gene signature, Survival prediction, Cancer

## Abstract

Prognosis prediction is difficult in hepatocellular carcinoma (HCC) due to high heterogeneity and complex etiology. It has recently been discovered that cuproptosis is a type of programmed cell death. However, its significance for HCC is still unclear. We analyzed mRNA expression profiles and clinical information from public databases to determine whether cuproptosis-related genes are associated with improved prognoses for HCC patients. The training cohort consisted of HCC patients from The Cancer Genome Atlas (TCGA), and the validation cohort relied on the International Cancer Genome Consortium (ICGC) database. We constructed a signature containing four genes using the least absolute shrinkage and selection operator (LASSO) COX regression model for calculating risk scores. Two risk groups were formed based on the median score. A significant improvement in survival was observed in the low-risk group compared to the high-risk. The multivariate Cox regression analysis showed that the risk score was an independent predictor of overall survival (OS). Further confirmation of the predictive accuracy of this signature is provided by receiver operating characteristic (ROC) analysis. Functional analysis revealed differences in immune status between the two risk groups. All the results described above were confirmed in the validation cohort. Therefore, a novel cuproptosis-related signature has the potential as a prognostic biomarker for HCC patients. Drugs developed to target cuproptosis-related genes may open up new pathways for treating HCC.

## 1 Introduction

There is a close link between cancer eradication and various modalities of cell death. For cancer cells that have resisted conventional therapy, ferroptosis induction has proved promising [1]. The journal Science recently published a study describing the discovery of a new method for copper-dependent cell death distinct from apoptosis and ferroptosis: cuproptosis [2]. The authors identified 10 regulatory genes in the research (positively regulated genes: FDX1, LIAS, LIPT1, DLD, CLAT, PDHA1, PDHB; negatively regulated genes: MTF1, CLS, CDKN2A). Cuproptosis is closely linked to protein lipoylation during tricarboxylic acid synthesis (TCA). They also found that cuproptosis played a role in distinguishing genetic divergence between individuals, as cuproptosis could be mediated by ferredoxin 1 and lipoic acid. Since dysregulation of copper homeostasis plays a significant role in cancer growth, angiogenesis, and metastasis [3], studying copper death holds promise for the development of new drugs to treat cancer. The copper metabolism in liver disease and hepatocellular carcinoma (HCC) continues to be investigated. Zhang et al. [4] reported that serum copper and ceruloplasmin levels could be used to detect the degree of cirrhosis and HCC. Koizumi et al. [5] proved that redox-active copper could initiate acute hepatitis and HCC. Copper oxide nanoparticles induced cytotoxicity and apoptosis in HepG2 cells, possibly through mitochondrial pathways and reactive oxygen species (ROS) [6, 7, 8]. According to the above studies, cuproptosis contributes to many liver diseases. The research about cuproptosis will bring new ideas for treating HCC.

A study of cuprotosis can also be useful for predicting the prognosis of other types of cancer. Based on the Gene Expression Omnibus (GEO) database and the TCGA database, Li et al. [9] identified oral squamous cell carcinoma (OSCC) differentially expressed genes. Their findings provide a new perspective on arecoline-related OSCC by demonstrating a link between arecoline, cuproptosis, Cancer-associated fibroblasts (CAFs), and OSCC metastasis. Ji et al. [10] reported that clinicopathological traits, prognosis, and elesclomol sensitivity were associated with patient molecular subtypes. Their findings might contribute to a more thorough understanding of cuproptosis-related genes and the novel treatment strategy for kidney renal clear cell carcinoma (KIRC). A variety of cancers have been extensively studied for cuproptosis-related genes, but hepatocellular carcinoma (HCC) have not. As a result of the limited treatment options for HCC, new therapeutic targets and target-based drugs must be developed. Several studies have examined cuprotosis-related genes and prognoses in HCC, but few have built prognostic models for the disease. Alterations in many cuproptosis-related genes may contribute to HCC progression, but methods to predict prognosis are currently lacking.

Our study involved the collection of mRNA expression profiles and clinical details from a public database of HCC patients. In addition, we developed a polygenic signature for cuproptosis based on data from the TCGA and ICGC cohorts. We found CDKN2A, GLS, DLAT, and LIPT1 were determined to be hub genes. Finally, we observed potential immune-related sympathies using functional enrichment analysis. We developed a gene signature to stratify patients to confirm further the prognostic value of cuproptosis-related genes in HCC patients. An intensive study of cuproptosis-related genes is expected to break new pathways for treating HCC.

## 2 Materials and methods

### 2.1 Data gathering

As a part of this study, RNA-sequencing (RNA-seq) data of 365 HCC patients were obtained from the TCGA (https://portal.gdc.cancer.gov/repository). Another 231 tumor samples were obtained from the ICGC (https://dcc.icgc.org/projects/LIRI-JP). We obtained a waiver from the institutional review board prior to conducting this study because the data utilized for this research were fully de-identified. The genes associated with cuproptosis were also retrieved using the TCGA and ICGC data access protocols.

### 2.2 Prognostic cuprotosis-related gene signature construction and validation

We identified DEGs in the TCGA based on a false discovery rate (FDR) of 0.05. By univariate Cox regression analysis, we determined the prognostic value of genes associated with cuproptosis. The STRING database (version 11.0) was employed to generate a protein-protein interaction network of prognosis-related DEGs based on which hub genes were identified [11]. LASSO-penalized Cox regression analysis was used to reduce overfitting [12, 13]. Fitting the LASSO model was performed using the Cox model, which is included in the R package glmnet. Using cross-validation, the shrinkage parameter was determined. Each patient's risk score was also calculated by normalizing each gene's expression levels and corresponding regression coefficients. According to their predicted risk scores, the training cohort was divided into high- and low-risk groups (with the median risk score used as a cutoff). We performed PCA with the prcomp function on the normalized data, and t-SNE on the Euclidean distance matrix. This study attempted to develop a robust prognostic gene signature for HCC using the LASSO Cox regression model. We performed a ten-fold cross-validation on the training set to calculate the weight of the LASSO penalty (denoted as λ). Four genes were identified for the signature based on the optimal value of λ (0.0102). The risk score was calculated as follows: e^(0.537^∗^expression level of LIPT1+0.388^∗^expression level of DLAT+0.059^∗^expression level of GLS+0.192^∗^expression level of CDKN2A)^. In order to evaluate the predictive power of gene signatures, survivalROC curves were analyzed using the “sevivalROC” R package.

### 2.3 Functional enrichment analysis

A comparison between high-risk and low-risk groups based on DEGs, Gene Ontology (GO) and Kyoto Encyclopedia of Genes and Genomes (KEGG) analyses.

### 2.4 Statistical analysis

Student's t-tests were used to compare gene expression between tumors and normal tissues, while Chi-square tests were used to determine proportions. We used BH-adjusted Mann-Whitney tests to compare immune cell or pathway ssGSEA scores between high-risk and low-risk groups; An analysis of Kaplan-Meier curves and a log-rank test were used to compare OS among the two groups. In order to determine independent predictors of OS, we conducted both univariate and multivariate analyses using the Cox regression model. The P < 0.05 significance level in this study was determined by two-tailed P values unless otherwise stated.

### 2.5 Ethics approval and consent to participate

The data in this article were obtained with permission from the TCGA and ICGC databases. Since the two are public databases, after being approved by our Ethics Committee, ethical approval and informed consent are waived.

## 3 Results

**Figure 1:**
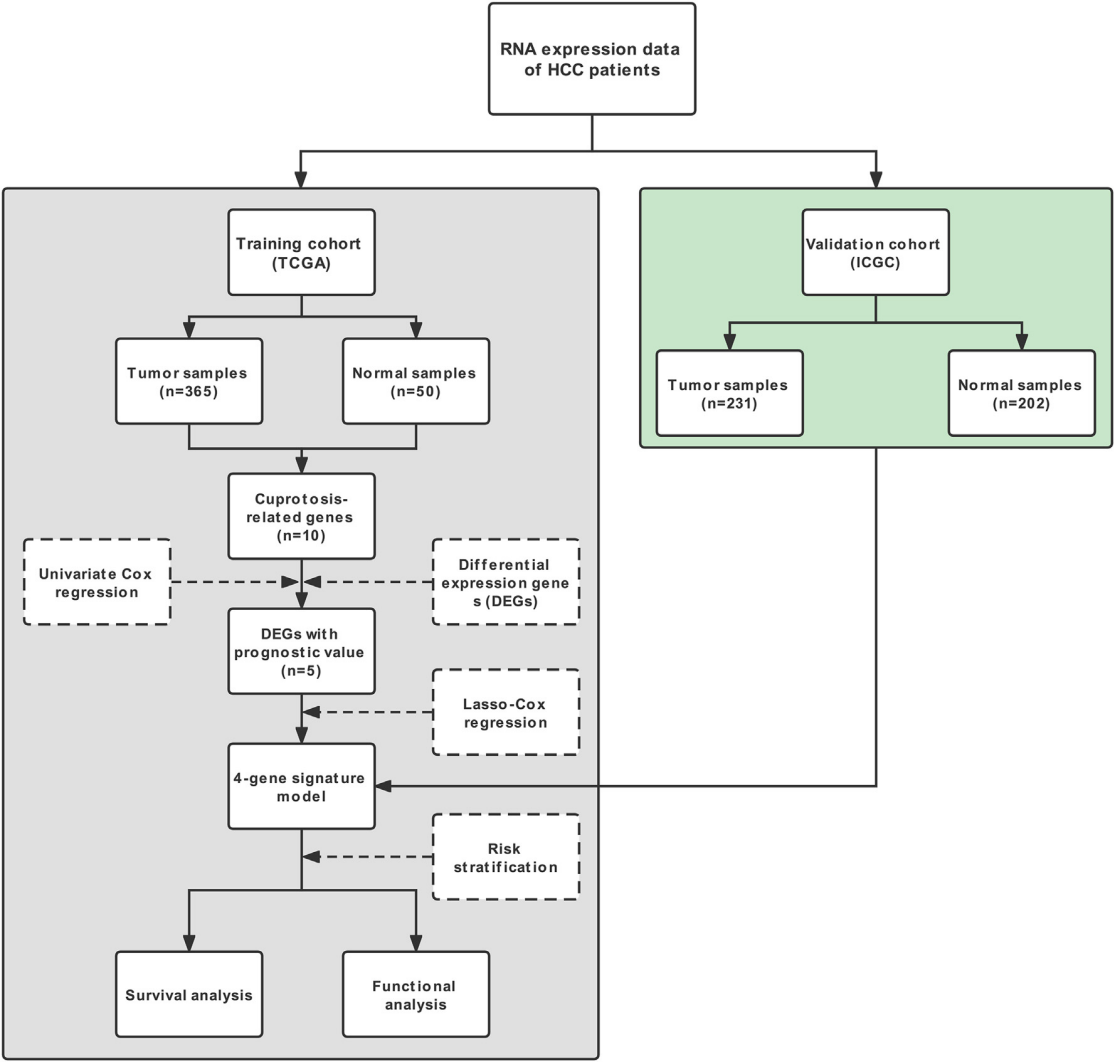
Flow chart of data collection and analysis.

In the TCGA cohort, 365 patients with HCC were studied, while in the ICGC cohort, 231 patients with HCC were studied. The clinical characteristics of the two groups were shown in Table 1. A flow chart of this study was shown in Figure 1.

**Table 1:** Clinical characteristics of the HCC patients used in this study.

	TCGA cohort	ICGC cohort
Number of patients	365	231
Survival status (%)		
Alive	235 (64.4%)	189 (81.8%)
Deceased	130 (35.6%)	42 (18.2%)
Gender (%)		
Female	119 (32.6%)	61 (26.4%)
Male	246 (67.4%)	170 (73.6%)
Grade (%)		
G1	55 (15.1%)	NA
G2	175 (47.9%)	NA
G3	118 (32.3%)	NA
G4	12 (3.3%)	NA
Stage (%)		
Stage I	170 (46.6%)	36 (15.6%)
Stage II	84 (23%)	105 (45.5%)
Stage III	83 (22.7%)	71 (30.7%)
Stage IV	4 (1.1%)	19 (8.2%)
OS days, median (IQR)	596 (344, 1088)	780 (510, 1095)
Age, median (IQR)	61 (52, 69)	69 (62, 74)

HCC, Hepatocellular carcinoma; TCGA, The Cancer Genome Atlas; ICGC, International Cancer Genome Consortium; OS, Overall survival; IQR, Interquartile range.

### 3.1 Analyze TCGA cohort DEGs for prognosis

**Figure 2:**
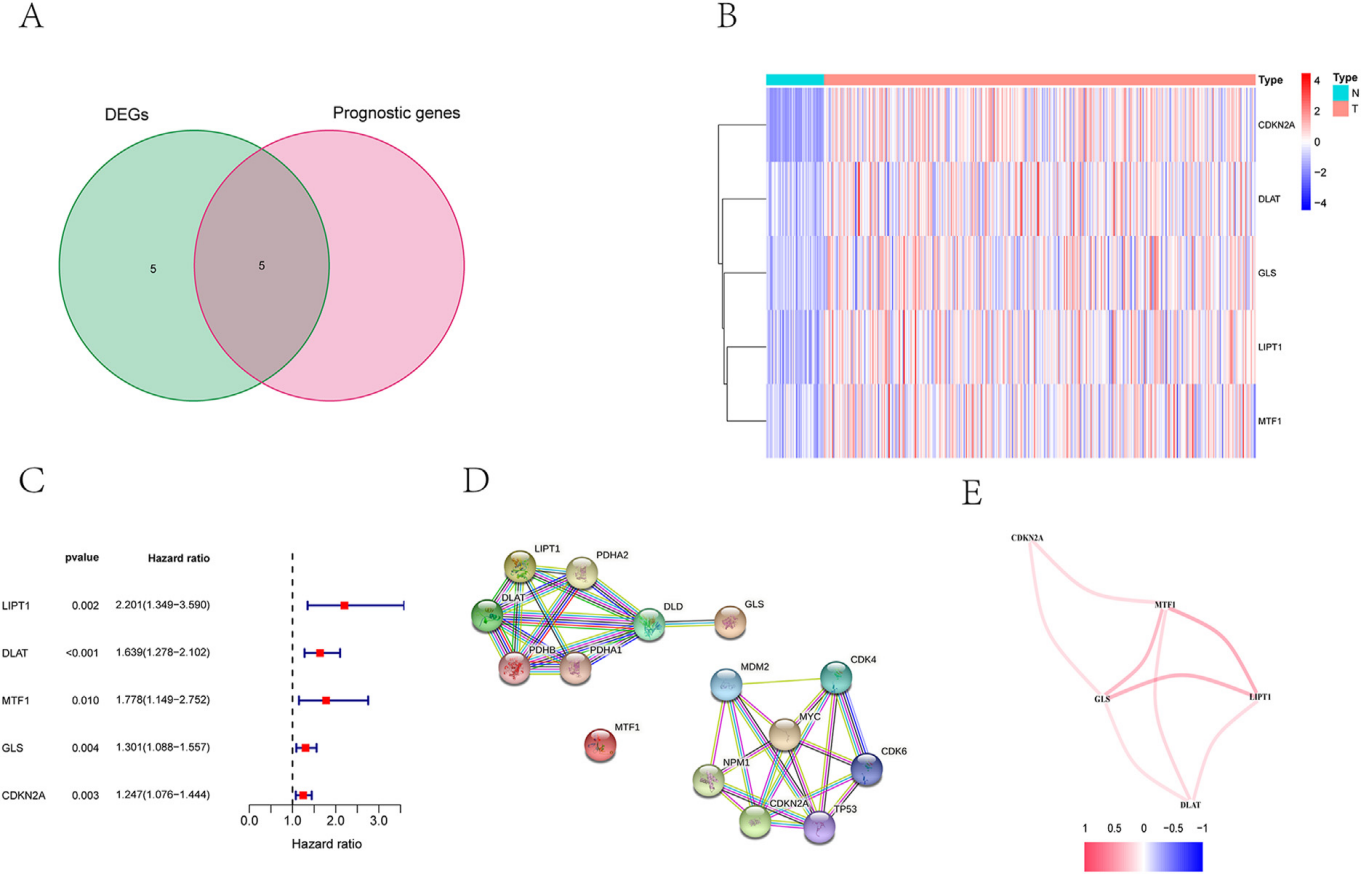
Candidate cuproptosis-related genes in the TCGA cohort. (A) To determine which genes are differentially expressed between tumors and adjacent normal tissue that are correlated with overall survival. (B) In tumor tissue, all 5 overlapping genes were upregulated. (C) These forest plots illustrate the results of the univariate Cox regression analysis between gene expression and OS. (D) As indicated by the PPI network downloaded from the STRING database, candidate genes interacted with each other. (E) The correlation network of candidate genes, colored according to the correlation coefficients.

The differential expression analysis revealed that all ten genes related to cuprotosis were differentially expressed in normal and HCC tissues. Five differentially expressed genes (DEGs) were associated with survival (Figure 2A). Figure 2B demonstrated the expression of the five prognosis-related DEGs in normal and tumor tissues. Figure 2C showed the univariate Cox regression analysis of the five prognosis-related DEGs. The increased risk of death in HCC patients is associated with high expression of these five genes. Among them, the LIPT1 gene had the highest hazard ratio of 2.201. The results of Figure 2D indicated close interactions among the cuproptosis-related genes, except for MTF1. We analyzed the correlations among the five DEGs to further investigate the target gene interactions. All target genes exhibited positive correlations, with MTF1, GLS, and LIPT1 showing the strongest correlations.

### 3.2 Constructing a prognostic model in the TCGA cohort

**Figure 3:**
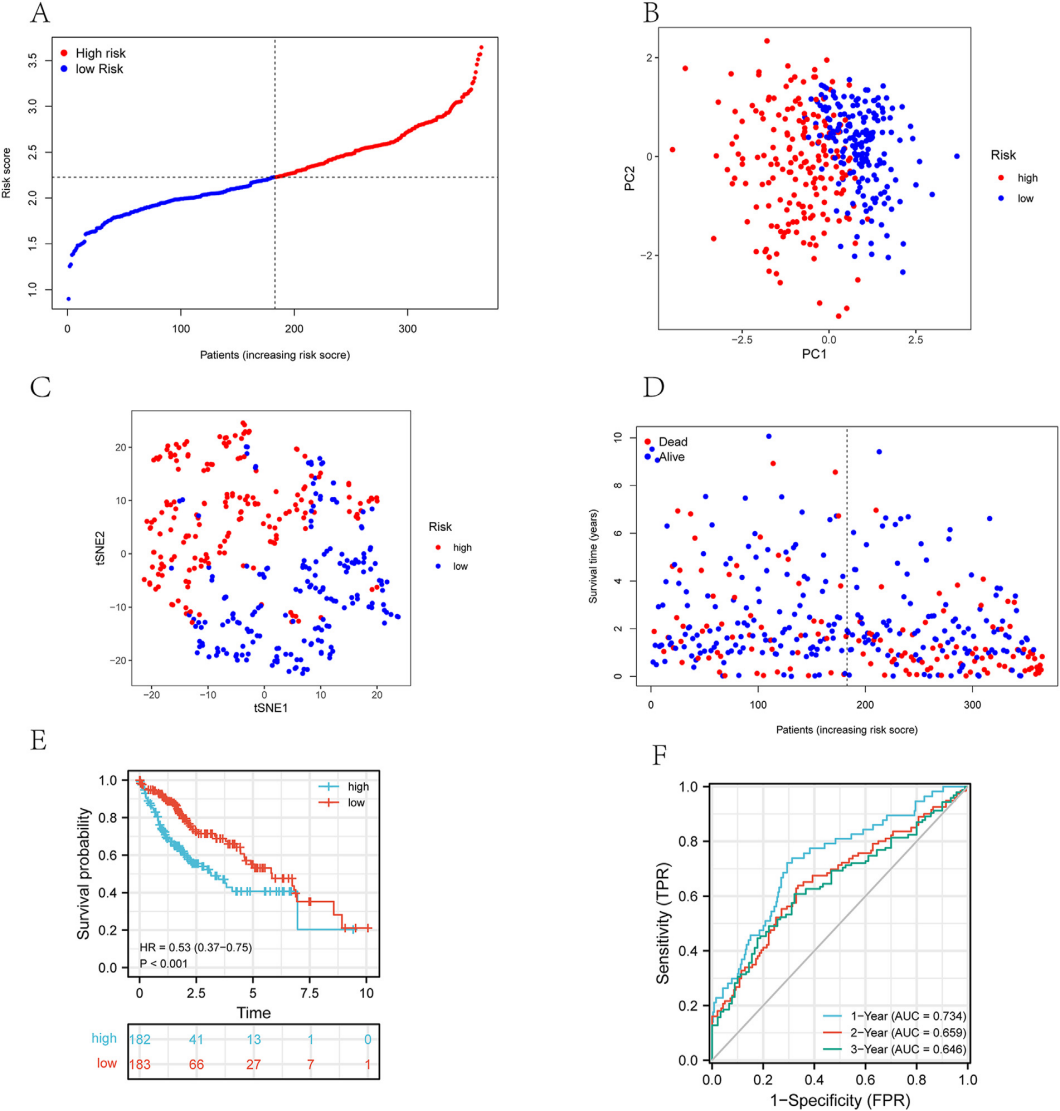
Analysis of the 4-gene signature model in the TCGA cohort. (A) The distribution and median risk scores among the TCGA cohort. (B) PCA plot of the TCGA cohort. (C) t-SNE analysis of the TCGA cohort. (D) The distributions of OS status, OS and risk score in the TCGA cohort. (E) Kaplan-Meier curves for the OS in the high-risk and low-risk groups of the TCGA cohort. (F) In the TCGA cohort, the AUC of time-dependent ROC curves proved that the risk score is a good prognostic indicator.

Our final four genes (LIPT1, DLAT, GLS and CDKN2A) were selected for signature construction using LASSO Cox regression model. The formula for calculating the risk score: e^(0.537^∗^expression level of LIPT1+0.388^∗^expression level of DLAT+0.059^∗^expression level of GLS+0.192^∗^expression level of CDKN2A)^. Patients were categorized into high-risk (n = 182) and low-risk (n = 183) groups by median score (Figure 3A). PCA and t-SNE were performed to evaluate grouping of samples. As a result of the high level of dispersion between the different risk groups, the grouping results were reliable (Figure 3B and C) and that each group had a significantly different death rate (Figure 3A–D). Compared to those with high-risk scores, those with low-risk scores had significantly longer OS (Figure 3E). Based on the receiver operating characteristic (ROC) curve, further clinical prognostic values were determined. According to the OS risk scores at 1 year, 2 years and 3 years, the areas under the curve (AUC) were 0.734, 0.660, and 0.646, respectively (Figure 3F).

### 3.3 A four-gene signature from the ICGC cohort was validated

**Figure 4:**
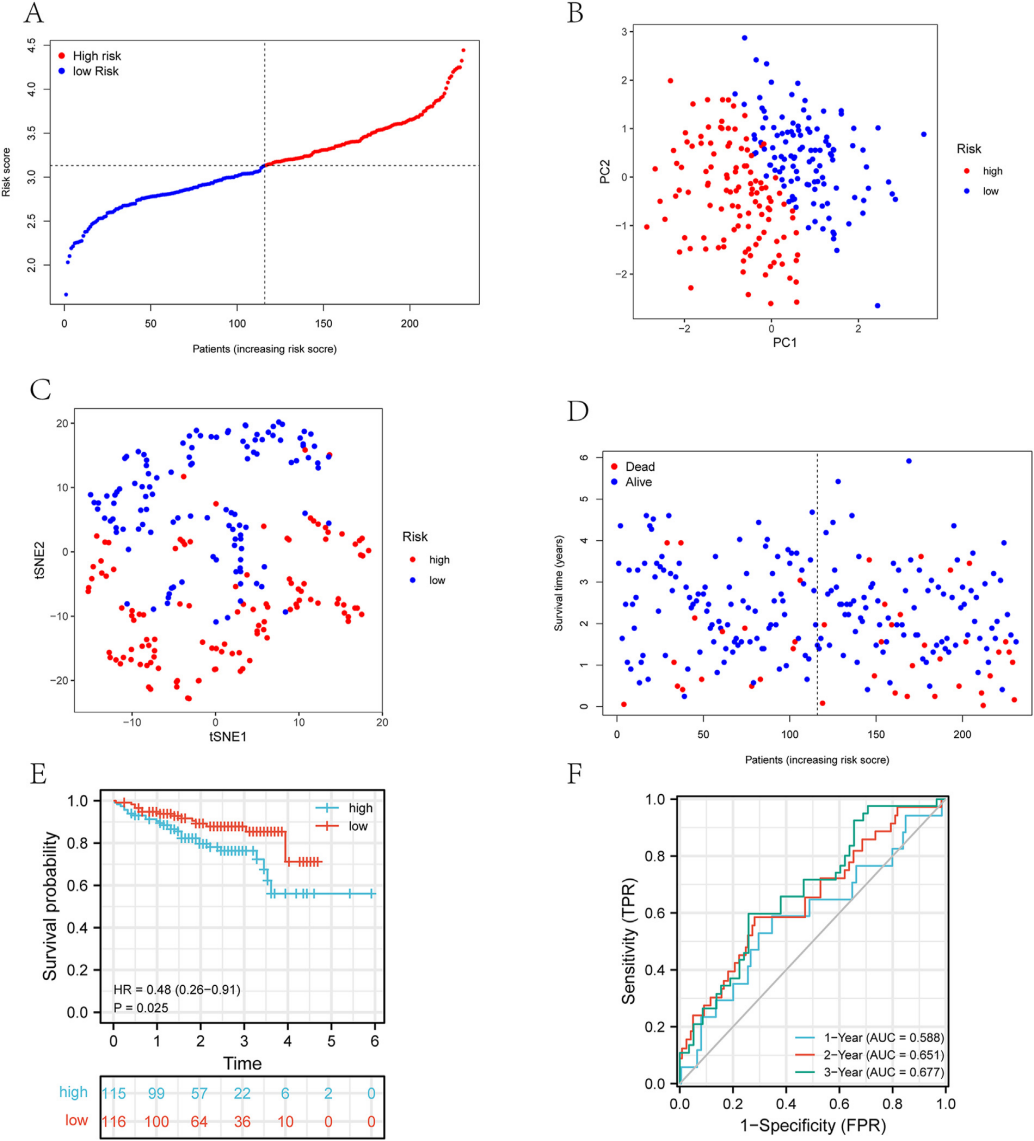
Analysis of the 4-gene signature model in the ICGC cohort. (A) The distribution and median values of the ICGC risk scores. (B) PCA plot of the ICGC cohort. (C) t-SNE analysis of the ICGC cohort. (D) The distributions of OS status, OS and risk score. (E) Graphs of Kaplan-Meier curves for patients in the high-risk group and the low-risk group. (F) AUC of time-dependent ROC curves in the ICGC cohort.

Gene signatures developed in the TCGA cohort were validated using the ICGC cohort (Figure 4A). Results from the ICGC cohort showed that patients were distributed differently between the PCA subgroup and the t-SNE subgroup (Figure 4B and C). High-risk groups had shorter survival times than low-risk groups (Figure 4D, P < 0.001), including more deaths (Figure 4E, P = 0.025). Additionally, the AUCs of the four-gene signature was 0.588 at 1 year, 0.651 at 2 years, and 0.677 at 3 years (Figure 4F). A 4-gene signature was found to be predictive in the training cohort and confirmed in the validation cohort.

### 3.4 The 4-gene signature was an independent risk factor for prognosis

**Figure 5:**
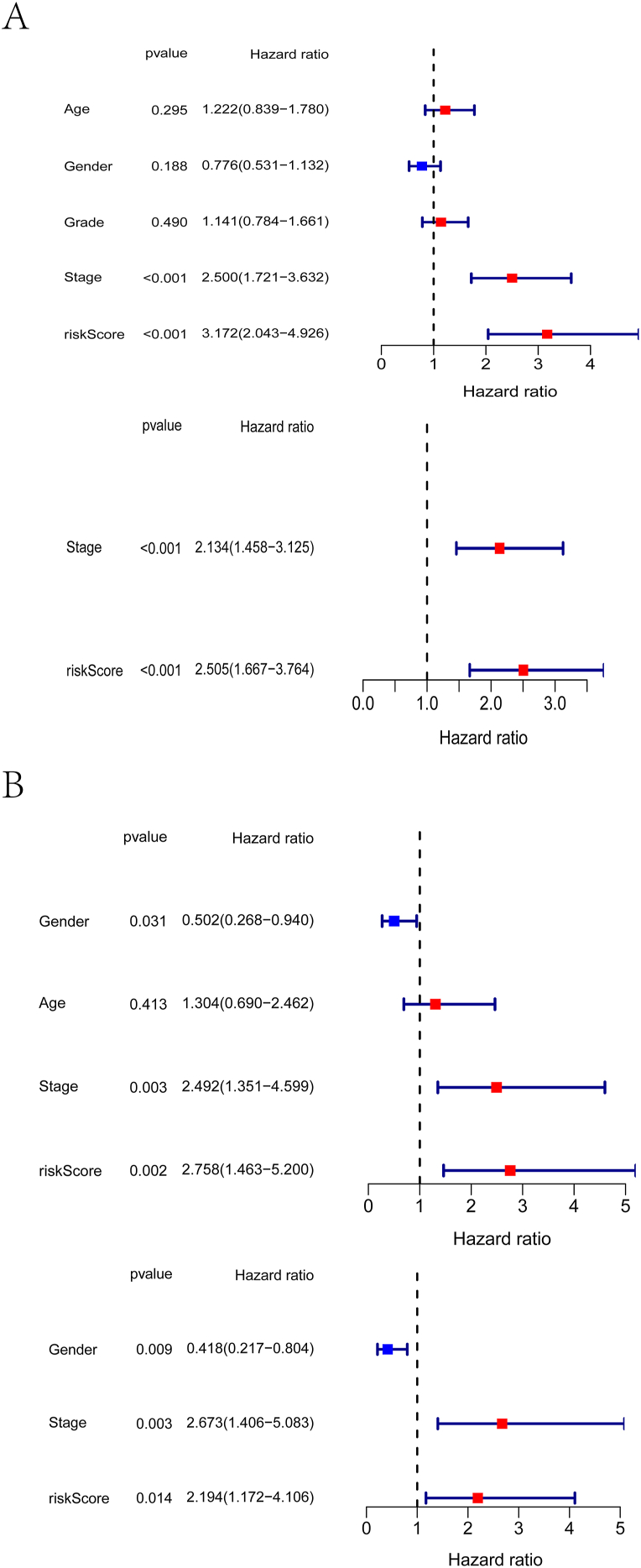
In the TCGA derivation cohort (A) and in the ICGC validation cohort (B), univariate and multivariate Cox regression analyses were performed.

To further explore whether our constructed 4-gene signature could predict patient survival independently of clinical characteristics, we performed a multivariate Cox regression analysis. Cox regression analysis of the univariate data showed significant associations between risk scores and OS in the TCGA and ICGC cohorts (HR = 3.172, 95% CI = 2.043–4.926, P < 0.001; HR = 2.758, 95% CI = 1.463–5.200, P = 0.002) (Figure 5A and B). The risk score was an independent predictor of OS in a multivariate Cox regression analysis with other confounding factors (TCGA cohort: HR = 2.505, 95% CI = 1.667–3.764, P < 0.001; ICGC cohort: HR = 2.194, 95% CI = 1.172–4.106, P = 0.014; Figure 5A and B). Results from the training and validation cohorts indicated that risk scores could predict patient survival independently of clinical characteristics.

### 3.5 Analysis of functional relationships in the TCGA and ICGC cohorts

**Figure 6:**
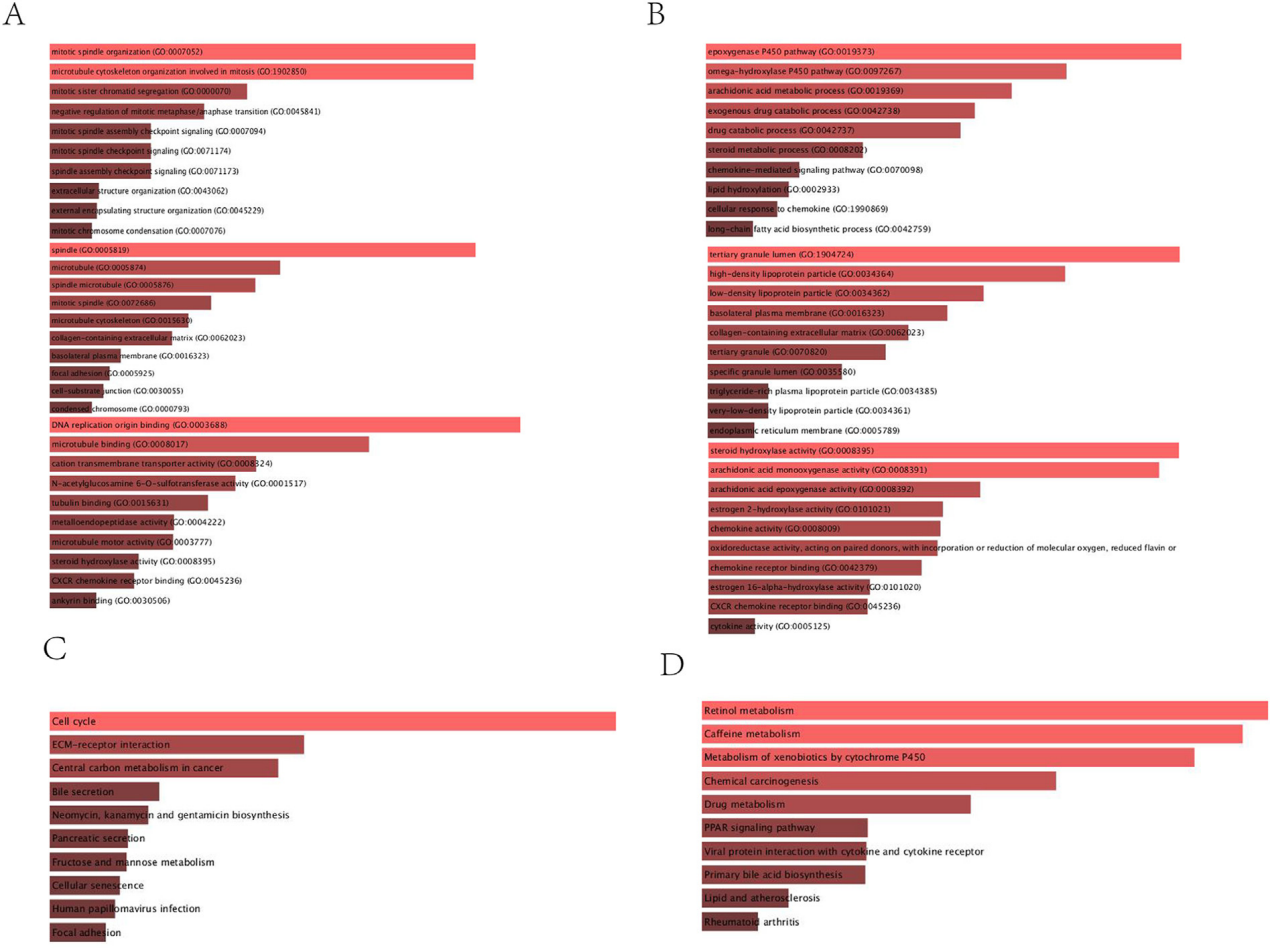
The results of GO (A, B) and KEGG analyses (C, D). GO enrichment (A, C) and KEGG pathways (B, D) are displayed for the TCGA cohort (A, C) and ICGC cohort (B, D).

We identified the biological functions and pathways associated with risk scores with GO enrichment and KEGG pathway analysis. In accordance with expectations, DEGs were associated with molecular functions (P. adjust < 0.05, Figure 6A and B). Immunological functions, such as the binding of the CXCR chemokine receptor, were evaluated. According to KEGG results of the TCGA and ICGA cohort, many immune-related pathways were discovered, such as mitotic spindle assembly checkpoint signaling, spindle assembly checkpoint signaling, chemical carcinogenesis, and viral protein interaction with cytokine and cytokine receptor (Figure 6C and D).

**Figure 7:**
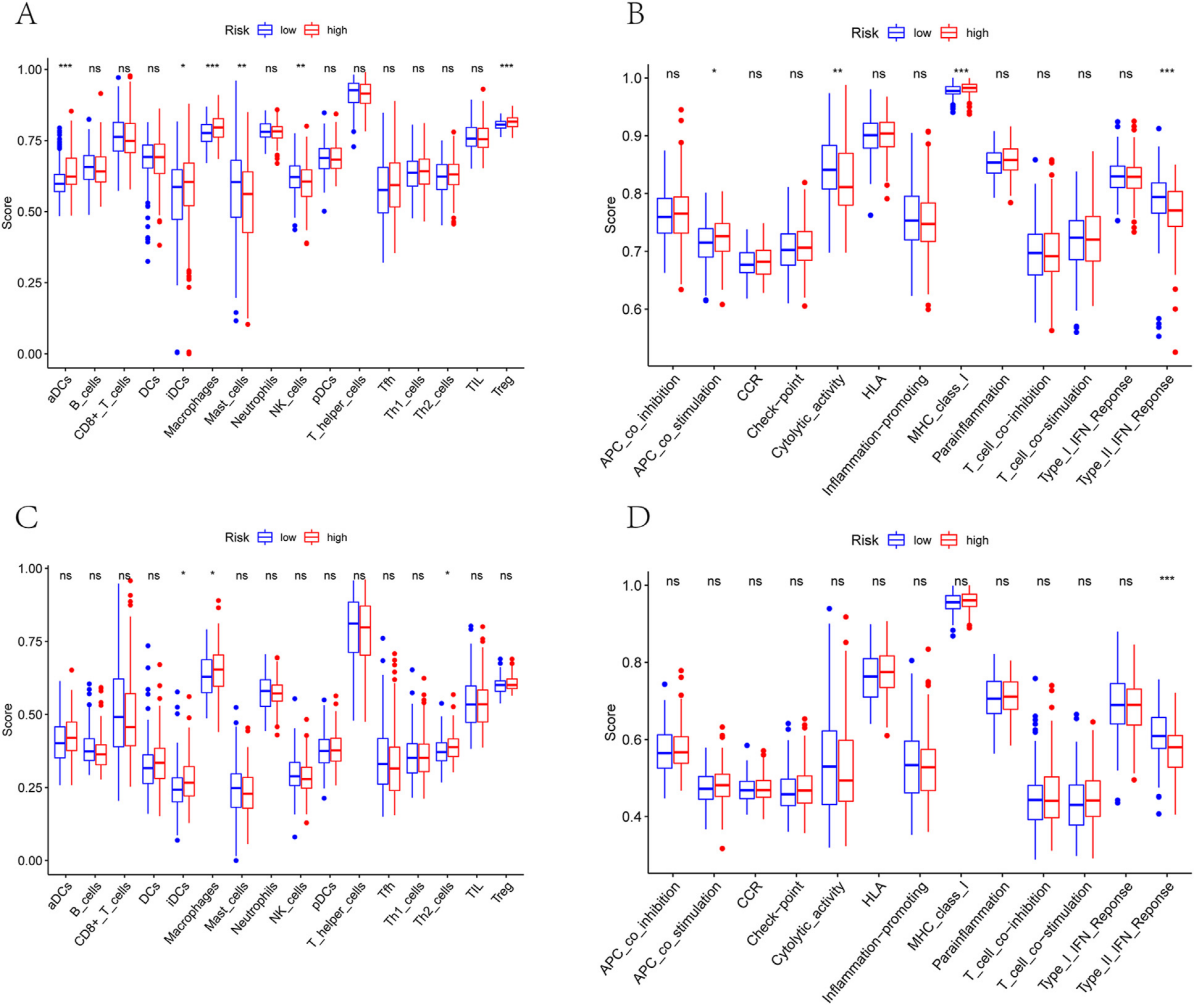
The ssGSEA scores in TCGA cohorts (A, B) and ICGC cohorts (C, D) between different risk groups. Boxplots show the scores of 16 immune cells (A, C) and 13 immune-related functions (B, D). CCR, cytokine–cytokine receptor. Adjusted *P* values were showed as: ns, not significant; ∗, *P* < 0.05; ∗∗, *P* < 0.01; ∗∗∗, *P* < 0.001.

The enrichment scores of immune cell subsets, function, and pathway categories were measured using ssGSEA in order to investigate the correlation between risk scores and immune status. Components of antigen presentation, such as ADCs, iDCs, and costimulatory scores, differ significantly. In the TCGA cohort, there was a difference in MHC class I scores between the low-risk and high-risk groups (P < 0.05, Figure 7A and B). Additionally, there were lower scores for NK cells, type II IFN response, and type I IFN response in the high-risk group compared with macrophages, Treg cells, and checkpoint activity (P < 0.05, Figure 7C and D). A comparison of ICGC cohorts confirmed the above conclusions in TCGA. A thorough review of the effects of type I and type II IFN on tumours and the efficacy of anti-type I IFN therapies has previously been published. Interferon-related immune dysfunction might contribute to increased mortality rates in high-risk patients.

## 4 Discussion

Several studies have demonstrated that copper could promote the growth and development of animals and improve their immune function. However, high levels of copper could also have some adverse effects on humans. There is a significant clinical need for effective HCC treatment strategies, and novel therapeutic targets and biomarkers remain a critical unmet need for HCC treatment.

Our study systematically examined the expression of cuproptosis-associated genes in HCC tumor tissue, and found five OS-related genes. In conclusion, four genes were selected to construct signatures based on LASSO-COX regression analysis. Functional analysis showed that immune-related pathways are enriched.

There is growing evidence that imbalanced copper homeostasis contributes to the development of tumors and causes irreversible damage to cells. The accumulation of reactive oxygen species, inhibition of the proteasome, and inhibition of angiogenesis are some of the mechanisms by which copper can cause multiple types of cell death, including apoptosis and autophagy. The results done by some researchers suggest that copper therapy is a potential anti-cancer strategy [14]. By combining copper with new biomaterials, copper can kill cancer cells effectively. Many researchers has suggested that cancer patients have higher levels of copper in serum and tumor tissue than healthy subjects [15]. In cancer progression, copper contributes to three fundamental processes: cell proliferation, angiogenesis, and metastasis [16]. In order to reduce the effect of copper on the body, it is currently possible to take Cu chelators [17], Tetrathiomolybdate (TM), D-penicillamine (D-pen) and trientine (Trien) are three such compounds [18, 19]. The prognostic model proposed in this research consisted of four genes (LIPT1, DLAT, GLS, CDKN2A) associated with cuproptosis.

There has been intense research into the mechanisms underlying cuproptosis in tumor cells in recent years. As a result of the DEG analysis, we found immune-related biological processes and pathways to be enriched in GO analysis. There may be a close connection between cuproptosis and tumor immunity. In the present study, we found differences in antigen-presenting cell expression between low-risk and high-risk groups. We, therefore, do not rule out that cuproptosis cells attract antigen-presenting cells (APCs) to perform their functions. A significant increase in macrophages and Treg cells was seen in both TCGA and ICGA cohorts, and their expression in HCC was associated with poor outcomes [20]. Additionally, on the basis of results from the type II IFN response, type I IFN response, and the fraction of NK cells, it can be speculated that a higher risk score would be associated with impaired immunity. Our data sources are public databases and lack our own experimental validation. At present, the prognosis of risk score and immune infiltration and related pathway expression is only in the theoretical stage, and further research is lacking.

In this study, we identified four genes associated with cuproptosis that can be used to develop a prognostic model. In training and validation cohorts, the model was shown to be independently associated with OS, providing a novel approach to predicting prognosis in HCC. However, there were some limitations to our study. First, the current study was based on public databases. Although we validated our findings in an independent validation cohort, a large sample of the prospective study cohort was still needed to increase the prognostic model's applicability further. Second, the results of this study showed a correlation between cuproptosis-related genes and the prognosis of patients with HCC, and further research is needed to explore the biological significance of this correlation. The underlying mechanisms between cuproptosis-related genes and tumor immunity in HCC remained poorly understood, and targeting cuproptosis-related genes might be a potential therapeutic strategy for HCC that warrants further investigation.

## Declarations

### Author contribution statement

Fan Yang: Conceived and designed the experiments; Performed the experiments; Analyzed and interpreted the data; Contributed reagents, materials, analysis tools or data; Wrote the paper.

Shitao Jiang, Yaoge Liu: Conceived and designed the experiments; Performed the experiments; Analyzed and interpreted the data; Contributed reagents, materials, analysis tools or data.

Ting Zhang, Chengpei Zhu, Lei Zhang: Conceived and designed the experiments; Performed the experiments; Wrote the paper.

Xinting Sang, Xin lu, Jiaxin Wei, Kaige Deng: Analyzed and interpreted the data; Contributed reagents, materials, analysis tools or data.

Yongchang Zheng, Yiyao Xu: Conceived and designed the experiments; Analyzed and interpreted the data.

### Funding statement

Yongchang Zheng was supported by Medical Sciences Innovation Fund for Medical Sciences (CIFMS) [2020-I2M-C&T-B-026], the Chinese Academy of Medical Sciences Innovation Fund for Medical Sciences (CIFMS) [2020-I2M-C&T-B-019], CHEN XIAO-PING Foundation for the development of science and technology of HUBEI province [CXPJJH1200008-10], WBE Liver Fibrosis Foundation [CFHPC 2020021], Natural Science Foundation of Beijing Municipality [Grant No. 7222130].

### Data availability statement

Data included in article/supp. material/referenced in article.

### Declaration of interest's statement

The authors declare no conflict of interest.

### Additional information

No additional information is available for this paper.
